# Transitioning from Soil to Host: Comparative Transcriptome Analysis Reveals the Burkholderia pseudomallei Response to Different Niches

**DOI:** 10.1128/spectrum.03835-22

**Published:** 2023-03-01

**Authors:** Ahmad-Kamal Ghazali, Mohd Firdaus-Raih, Asqwin Uthaya Kumar, Wei-Kang Lee, Chee-Choong Hoh, Sheila Nathan

**Affiliations:** a Department of Biological Sciences and Biotechnology, Faculty of Science and Technology, Universiti Kebangsaan Malaysia, Bangi, Selangor, Malaysia; b Department of Applied Physics, Faculty of Science and Technology, Universiti Kebangsaan Malaysia, Bangi, Selangor, Malaysia; c Institute of Systems Biology, Universiti Kebangsaan Malaysia, Bangi, Selangor, Malaysia; d Codon Genomics Sdn. Bhd., Seri Kembangan, Selangor, Malaysia; Cinvestav-IPN

**Keywords:** *Burkholderia pseudomallei*, comparative transcriptomics, adaptation, survival, virulence factors

## Abstract

Burkholderia pseudomallei, a soil and water saprophyte, is responsible for the tropical human disease melioidosis. A hundred years since its discovery, there is still much to learn about B. pseudomallei proteins that are essential for the bacterium’s survival in and interaction with the infected host, as well as their roles within the bacterium’s natural soil habitat. To address this gap, bacteria grown under conditions mimicking the soil environment were subjected to transcriptome sequencing (RNA-seq) analysis. A dual RNA-seq approach was used on total RNA from spleens isolated from a B. pseudomallei mouse infection model at 5 days postinfection. Under these conditions, a total of 1,434 bacterial genes were induced, with 959 induced in the soil environment and 475 induced in bacteria residing within the host. Genes encoding metabolism and transporter proteins were induced when the bacteria were present in soil, while virulence factors, metabolism, and bacterial defense mechanisms were upregulated during active infection of mice. On the other hand, capsular polysaccharide and quorum-sensing pathways were inhibited during infection. In addition to virulence factors, reactive oxygen species, heat shock proteins, siderophores, and secondary metabolites were also induced to assist bacterial adaptation and survival in the host. Overall, this study provides crucial insights into the transcriptome-level adaptations which facilitate infection by soil-dwelling B. pseudomallei. Targeting novel therapeutics toward B. pseudomallei proteins required for adaptation provides an alternative treatment strategy given its intrinsic antimicrobial resistance and the absence of a vaccine.

**IMPORTANCE** Burkholderia pseudomallei, a soil-dwelling bacterium, is the causative agent of melioidosis, a fatal infectious disease of humans and animals. The bacterium has a large genome consisting of two chromosomes carrying genes that encode proteins with important roles for survival in diverse environments as well as in the infected host. While a general mechanism of pathogenesis has been proposed, it is not clear which proteins have major roles when the bacteria are in the soil and whether the same proteins are key to successful infection and spread. To address this question, we grew the bacteria in soil medium and then in infected mice. At 5 days postinfection, bacteria were recovered from infected mouse organs and their gene expression was compared against that of bacteria grown in soil medium. The analysis revealed a list of genes expressed under soil growth conditions and a different set of genes encoding proteins which may be important for survival, replication, and dissemination in an infected host. These proteins are a potential resource for understanding the full adaptation mechanism of this pathogen. In the absence of a vaccine for melioidosis and with treatment being reliant on combinatorial antibiotic therapy, these proteins may be ideal targets for designing antimicrobials to treat melioidosis.

## INTRODUCTION

Burkholderia pseudomallei is an environmental Gram-negative bacteria and the causative agent of melioidosis in humans. It is found in endemic tropical countries such as Malaysia, Thailand, Singapore, and Northern Australia (1, 2). The global burden of human melioidosis is predicted at 165,000 cases and 89,000 deaths annually, and in Malaysia, it is estimated that more than 2,000 patients die each year (3). Environmental exposure through cuts and abrasions, inhalation, or ingestion of bacteria-contaminated soil or water can result in human infection (4). Melioidosis cases have been frequently reported in farmers and agriculture workers, suggesting that their frequent contact with soil and standing water is responsible for the high number of cases. The disease has a wide range of clinical manifestations, including sepsis, bacteremia, pneumonia, and abscesses in multiple organs (2). The mortality rate varies by geography, ranging from 19% in Australia and up to 50% in northeast Thailand, and was recently estimated at ~50% of infected individuals globally (5, 6). This disease necessitates long-term intravenous and oral antibiotic treatments. Even though it is uncommon, a relapse of infection may occur if the initial infection is not completely eradicated (7).

When bacteria infect humans, they often encounter environments that are quite different from the natural environmental niches to which they are well adapted. B. pseudomallei has a unique trait that few bacteria possess: it can adapt to and thrive in a broad range of severe environmental niches. B. pseudomallei, which naturally reside in soil or water, experience large differences in temperature and available nutrients, as well as the presence of antibiotic and immune selective pressures in infected hosts, including amoeba, animals, plants, and humans ([Bibr B8][Bibr B9] 10). B. pseudomallei can persist in a low-nutrient environment (for example, in distilled water for 17 years) (8, 11), low oxygen content (12), acidic environments (13), and a wide temperature range (25°C to 42°C) for long periods of time (14). The ability of B. pseudomallei to adapt and survive in a variety of harsh environments may be due to its genome content and the activation of protein-encoding genes which protect it from environmental harm. The B. pseudomallei genome is large, consisting of two chromosomes (4.07 Mb and 3.17 Mb) (15). Chromosome 1 (Chr1) is engaged in core metabolism and cell growth, while chromosome 2 (Chr2) is involved in accessory functions related to adaptability and survival. The highly variable accessory genome across isolates may confer additional advantages for survival and replication in specific niches, including humans and the bacterium’s natural habitat of soil and surface water.

The factors which allow B. pseudomallei to survive in harsh environments remain poorly understood. A number of studies have previously reported on B. pseudomallei adaptation to different *in vitro* growth conditions (12, 16–18) and during infection of a host model (19, 20). Ooi et al. (16) constructed a B. pseudomallei “transcriptional condition compendium” representing transcriptional data from over 80 different situations. The data within the compendium support the proposition that genes in Chr2 are involved in accessory functions because these genes exhibit mosaic expression and are robustly expressed in a condition-dependent manner. Adaptation and survival of B. pseudomallei in the host during infection has mainly been studied using cell lines. B. pseudomallei virulence factors such as the type III secretion system (T3SS), type VI secretion system (T6SS), flagella, and capsular polysaccharide (CPS) all play significant roles in its adaptability and defense against the host immunological response (19, 20). Nonetheless, these studies utilized B. pseudomallei grown under standard laboratory conditions to infect the model host, and their findings may not truly represent actual changes in terms of proteins expressed during the transition from a soil-based environment to a whole-animal infection model.

In this study, we used well-described soil-like conditions (21) and a mouse model of melioidosis (22) infected with a local clinical isolate, B. pseudomallei UKMD286, to understand how B. pseudomallei adapts when challenged with a shift from soil to a mammalian host. Bacteria were grown in soil extract medium (SEM) (21) to mimic B. pseudomallei in a soil environment and were then used to infect mice. Illumina next-generation sequencing (NGS) technology was used to sequence RNA isolated from B. pseudomallei UKMD286 cultivated in SEM and from bacteria isolated from the organs of infected mice, and the reads generated were mapped to the B. pseudomallei UKMD286 genome (23). To aid data comparison across published studies, differentially expressed genes are reported based on B. pseudomallei K96243 gene homologs.

## RESULTS

### Mouse model of *B. pseudomallei* UKMD286 cultured in SEM infection.

**FIG 1:**
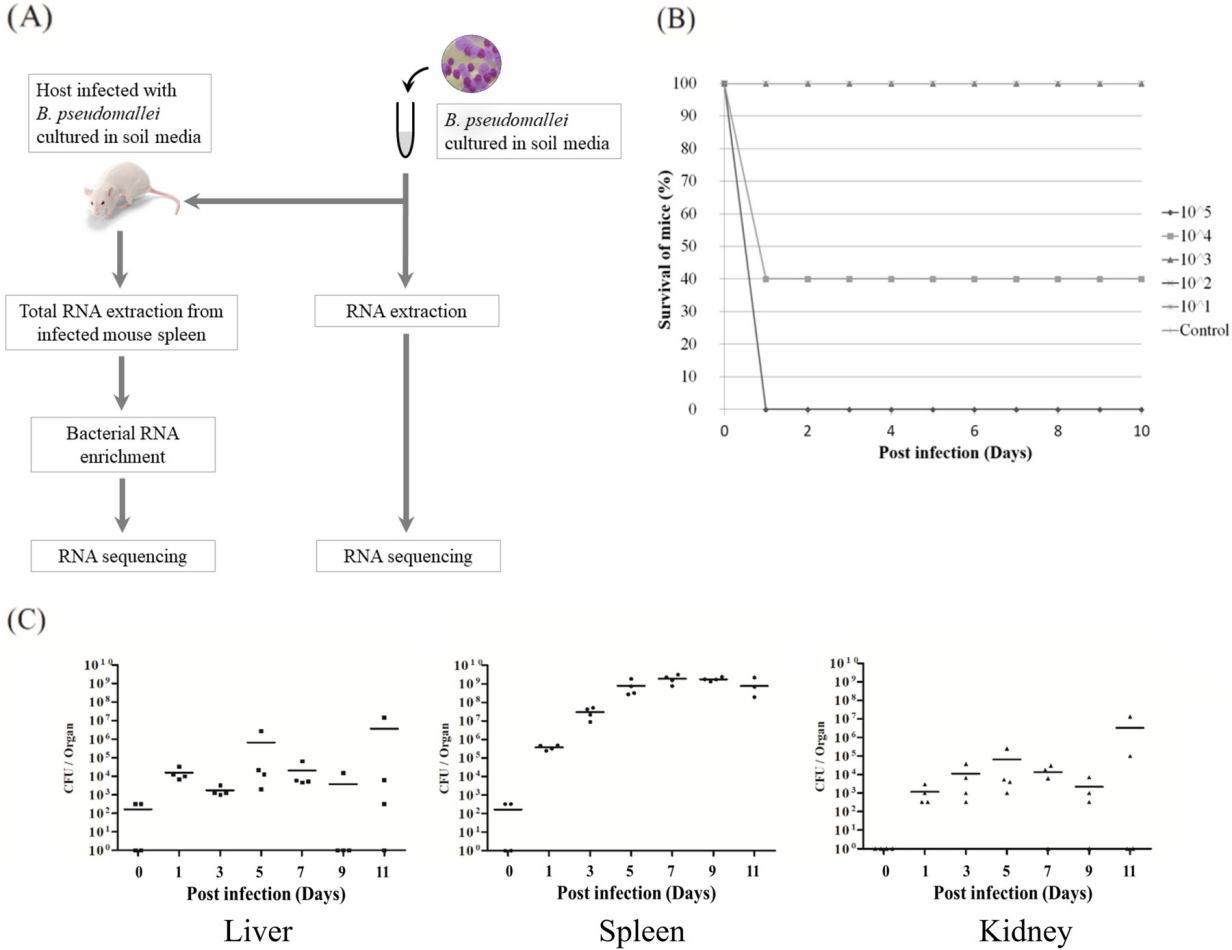
(A) Experimental design and steps to create the RNA-sequencing libraries. (B) Results of 50% lethal dose (LD_50_) analysis of Burkholderia pseudomallei UKMD286 cultured in soil extract medium SEM. Each group of mice (*n* = 5) was infected with 10^1^ to 10^5^ CFU UKMD286 through the intraperitoneal route. A group of mice were given phosphate-buffered saline (PBS) as control. Mice were monitored daily for signs and symptoms of disease. (C) Bacterial loads in BALB/c mice. Bacterial loads in the (left graph) livers, (center graph) spleens, and (right graph) kidneys of BALB/c mice at days 0, 1, 3, 5, 7, 9 and 11 after intraperitoneal infection with 0.1× LD_50_ CFU of B. pseudomallei UKMD286 cultured in SEM. Each point represents the CFU count for an individual mouse; horizontal lines indicate the geometric mean for each time point.

To determine changes in gene expression when B. pseudomallei is shifted from the soil environment into the *in vivo* host environment, B. pseudomallei cultured in SEM was used to establish a mouse infection model as previously described (22) (Fig. 1A). Our initial attempt to recoup bacteria from infected organs for bacterial RNA preparation (20) was unsuccessful; therefore, total RNA (mouse and bacterial RNA) from infected mouse spleen was prepared for a dual-RNA sequencing approach. However, a challenging and limiting factor of this approach is that bacterial RNA generally comprises less than 1% of the total RNA obtained from infected mouse organs (24). Hence, it was important to first determine the dose and time point at which the intracellular B. pseudomallei present in the organs were in the mid-log phase. This would determine the optimal dose to infect the mice and the optimal time point to harvest the organs postinfection.

First, we determined the 50% lethal dose (LD_50_) of B. pseudomallei UKMD286 cultivated in SEM to use when challenging BALB/c mice through the intraperitoneal (IP) route. As expected, increasing the infective dose correlated with increasing severity of the phenotypes (lethargic, ruffled fur, and paresis of both hind legs in the late stage of infection prior to succumbing to infection) typically observed in a melioidosis mouse model. Mice injected with 10^1^ and 10^2^ CFU of UKMD286 showed no symptoms and survived the 10-day observation period. Mice infected with >10^3^ CFU showed signs of decreased activity, rough hair coat, and hind limb paresis. Mice infected with 10^5^ CFU showed almost immediate signs of reduced activity and died within 24 h of infection (Fig. 1B). After 10 days postinfection (dpi), the LD_50_ value was calculated as 3.00 × 10^4^ CFU.

Based on the calculated LD_50_ dose, we infected BALB/c mice with 0.1 × LD_50_ B. pseudomallei UKMD286 (22) and monitored the kinetics of the bacterial load in various organs in BALB/c mice over the course of infection. For the transcriptome sequencing, it was necessary to enrich bacterial RNA from the total RNA extracted from infected mouse organs. Thus, it was essential to harvest the organs when bacterial counts were highest and at the mid-log phase. A total of 30 mice were challenged with 3.00 × 10^3^ CFU B. pseudomallei UKMD286. Bacterial loads were enumerated in infected mouse livers, spleens and kidneys every 48 h over 10 days (Fig. 1C). The infection resembled systemic acute melioidosis, as organs contained high bacterial loads as early as 24 h postinfection, with the highest burden in the spleen followed by the liver and kidney. Bacterial loads in the liver and kidney were low and inconsistent over the course of the 10-day infection (Fig. 1C).

From day 1 to day 3 postinfection, a low bacterial load was observed in the spleen. During this period, B. pseudomallei is most likely adapting to changes in the environment as it shifts from soil-like conditions to the host environment. These changes include differences in pH and temperature as well as the presence of reactive oxygen species (ROS) and the host’s initial immune response toward bacterial invasion. The bacterial loads subsequently increased significantly from days 3 to 7 postinfection, achieving ~10^9^ CFU per spleen. Over this period, the presence of actively dividing B. pseudomallei suggests that the bacteria had successfully adapted to the stresses of the new environment. From day 7 postinfection until the end of the experiment, bacterial growth slowed to a plateau. The high numbers of B. pseudomallei in the spleen confirmed that systemic acute melioidosis had successfully developed in BALB/c mice, and day 5 was selected as the time point which best reflected the active infection stage. Spleens were harvested from infected mice on day 5 postinfection for RNA preparation and bacterial gene expression profiling.

The initial approach used to prepare RNA from B. pseudomallei present in the infected organs was to isolate the live bacteria using a differential lysis and centrifugation method (20). However, we were unable to obtain intact RNA and opted to prepare whole-organ total RNA and perform dual RNA-sequencing on the mixed-population RNA. Spleens were homogenized and total RNA was prepared with RNA integrity numbers (RIN) greater than 8.0. Replicate samples of total RNA (mouse + bacteria) were labeled as M4 and M7 (Table 1). B. pseudomallei UKMD286 was also cultivated in SEM to mimic B. pseudomallei in its natural soil environment, and the high-quality replicate RNA samples (RIN > 9.0) prepared from the cultures are here referred to as FB2 and FB5.

**TABLE 1:** Statistics of sequence reads mapped to the Burkholderia pseudomallei UKMD286 genome

B. pseudomallei sample type	Sample ID	Total no. of reads (pre-processed)	Reads mapped to genomes (% reads mapped)	
			UKMD286 genome	Mouse genome (GRCm38)^*b*^
Cultured in SEM	FB2	12,687,174	12,487,278 (98.42%)	-
	FB5	17,241,728	16,214,697 (94.04%)	-
From infected spleens	M4	102,013,708	28,394 (0.03%)	95,137,125 (93.25%)
	M7	100,043,494	52,341 (0.05%)	94,726,644 (94.69%)
From infected spleens (enriched)	M4-En^*a*^	68,728,968	370,790 (0.54%)	56,929,823 (82.83%)
	M7-En^*a*^	180,714,288	422,822 (0.23%)	152,317,666 (84.29%)

a M4-En and M7-En refer to RNA derived from M4 and M7 samples, respectively, after rRNA depletion.

b To compare the bacterial-host RNA ratio, sequence reads from M4, M7, M4-En, and M7-En samples were also mapped to the mouse genome.

### *B. pseudomallei* genome-wide gene expression analysis during infection.

**FIG 2:**
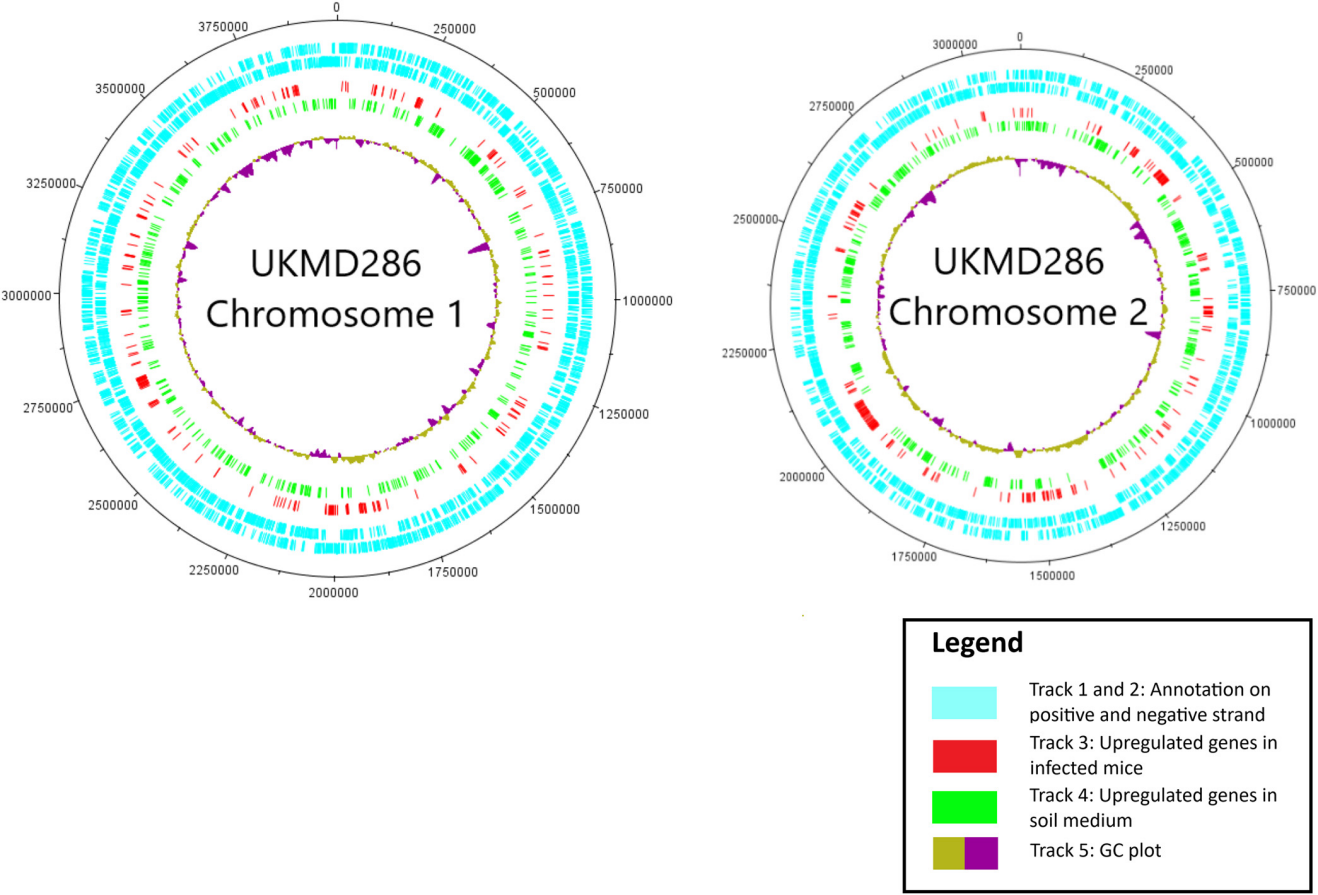
Circular map depicting expression of B. pseudomallei transcripts in FPKM (fragments per kilobase per million) mapped to the B. pseudomallei UKMD286 reference genome. Rings are labeled as tracks 1 (outermost) to 5 (innermost). Track 1: B. pseudomallei UKMD286 chromosome 1 (Chr1) or Chr2 annotation (positive strand). Track 2: B. pseudomallei UKMD286 Chr1 or Chr2 annotation (negative strand). Track 3: upregulated genes in infected mice. Track 4: upregulated genes in soil medium. Track 5: GC plot.

To guarantee high-quality expression data, dual RNA-sequencing normally requires a reasonably large amount of input RNA due to high rRNA (>98%) and low bacterial RNA content in organs harvested from infected animals (24). To acquire bacterial mRNA reads from within the population of infected mouse total RNA, the M4 and M7 samples were subjected to deep sequencing at 100 million reads coverage. Nonetheless, this strategy only captured a small fraction of RNA reads that were successfully mapped to the B. pseudomallei genome (0.03% to 0.05%). The M4 and M7 total RNA were treated to rRNA depletion to enrich the bacterial mRNA content and the M4-En and M7-En samples were subjected to deep sequencing. The coverage of bacterial reads mapped increased by up to 18-fold (0.23% to 0.54%, Table 1) when mapped to the B. pseudomallei UKMD286 genome (23). According to Rey et al. (25), a total of 300,000 bacterial reads should capture 90% of the bacterial transcriptome profile and is sufficient to assess gene expression between samples, and the number of reads from the enriched samples surpassed this. The differential expression profiles of B. pseudomallei UKMD286 cultured under both host (infection) and soil conditions are shown as a circular map according to the gene location on B. pseudomallei UKMD286 chromosomes 1 and 2 (Fig. 2).

### *B. pseudomallei* transcriptome changes when the bacteria shift from soil into the host.

The day 5 postinfection intracellular B. pseudomallei gene expression profile was compared with the transcriptome of bacteria grown in SEM. Statistical analysis (*P* < 0.01) combined with a 2-fold variation cutoff indicated that 1,434/6,096 (24%) B. pseudomallei UKMD286 genes were modulated under both infection and soil conditions (Supplemental File 1, Data Set S1). From this point onwards, genes positively regulated (induced) during infection relative to transcript levels for the bacteria grown in SEM are represented as genes induced during infection. Meanwhile, bacterial genes negatively regulated during mouse infection are presented as genes that are induced when the bacteria are grown in SEM.

**FIG 3:**
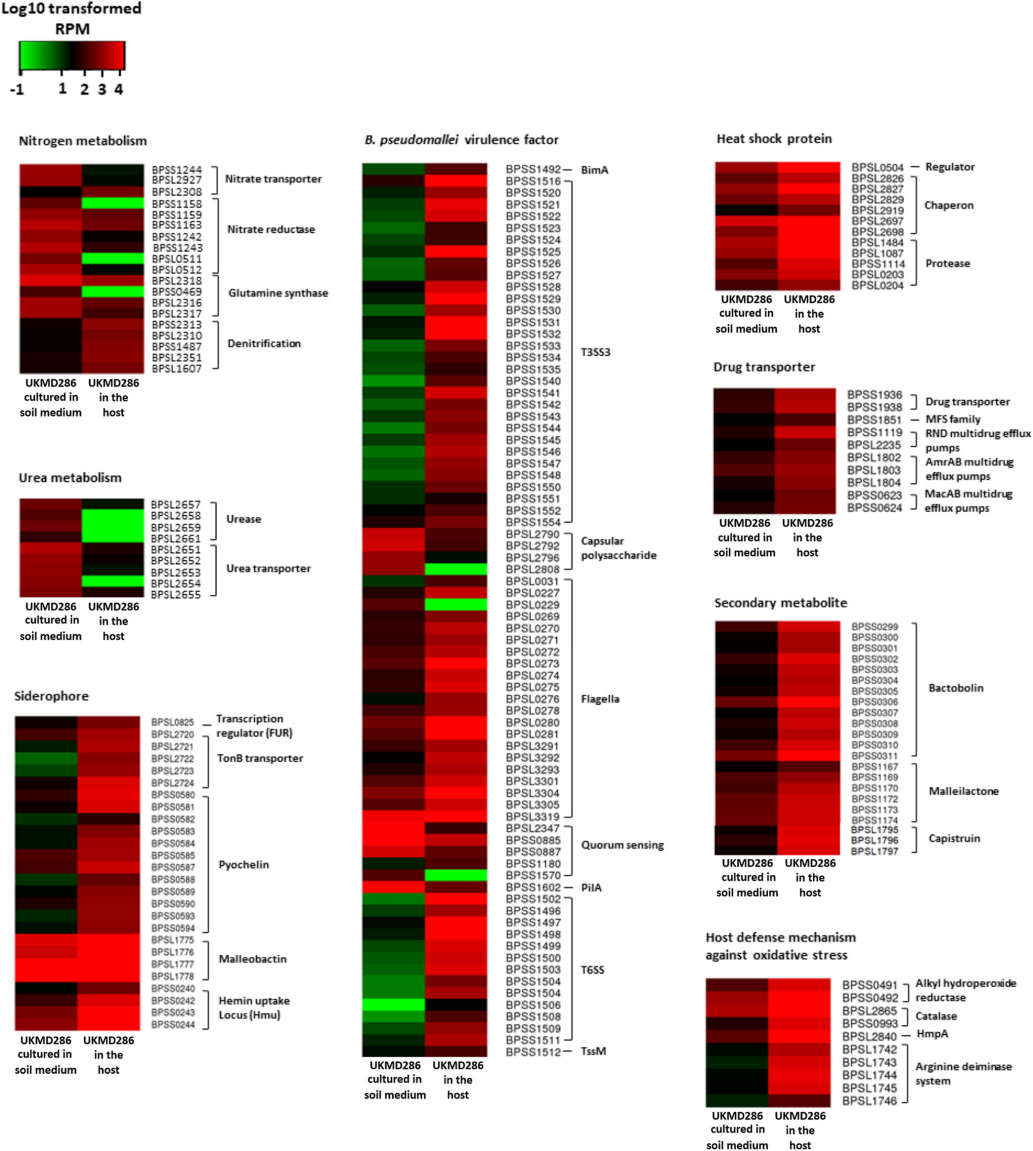
UKMD286-induced transcriptional responses in SEM and host environment. Heat maps of B. pseudomallei UKMD286 expression profiles in the host and in soil medium based on functional categories. Colors symbolize transformed FPKM counts, with green representing low expression, black representing intermediate expression, and red representing high expression.

Of a total of 1,434 B. pseudomallei genes, 959 (67%) were induced when the bacteria were grown in SEM (negatively regulated during mouse infection). Genes activated under SEM conditions encoded proteins important in metabolism and transportation (Fig. 3). Approximately 44% of the genes induced under SEM conditions were annotated as hypothetical or encoding proteins of unknown function but were assumed to play a role in soil adaptation (Supplementary File 1, Data Set S2). Gene function enrichment analysis of the positively regulated genes was then performed to further categorize them into biological functions. Based on the statistical analysis of KEGG biochemical pathways, the overexpressed genes were enriched for nitrogen and galactose metabolism in the bacteria cultured in SEM (Table 2). The remaining 475 out of 1,434 (23%) differentially expressed genes were induced during active infection of the mouse. The genes induced in the enriched *in vivo* samples encoded proteins engaged in pathogenesis, protein folding, motility, and responses to heat and stress (Fig. 3). Gene function enrichment analysis of the positively regulated genes within KEGG biochemical pathways demonstrated that the number of genes involved in cellular functions such as flagella assembly, oxidative phosphorylation, and the two-component systems was significantly higher during intracellular infection (Table 2).

**TABLE 2:** Enriched KEGG pathway analysis of Burkholderia pseudomallei when exposed to soil and host ecological niches

Environment	Functional category	KEGG pathway	Counts	Pop hits	*P*
Soil	Metabolism	bps00910: Nitrogen metabolism	15	39	1.01E−04
		bps00052: Galactose metabolism	7	16	8.65E−03
		bps00564: Glycerophospholipid metabolism	8	26	3.17E−02
		bps00500: Starch and sucrose metabolism	8	27	3.84E−02
	Transporter and other functions	bps02010: ABC transporters	44	217	4.19E−04
		bps03070: Bacterial secretion system	22	95	3.84E−03
		bps02020: Two-component system	27	137	1.17E−02
Host	Virulence factor	bps02040: Flagellar assembly	14	37	4.94E−06
	Metabolism	bps01053: Biosynthesis of siderophore group nonribosomal peptides	5	7	1.15E−03
		bps00910: Nitrogen metabolism	9	39	1.53E−02
		bps00190: Oxidative phosphorylation	12	69	3.01E−02
	Other functions	bps02020: Two-component system	21	137	1.05E−02

**FIG 4:**
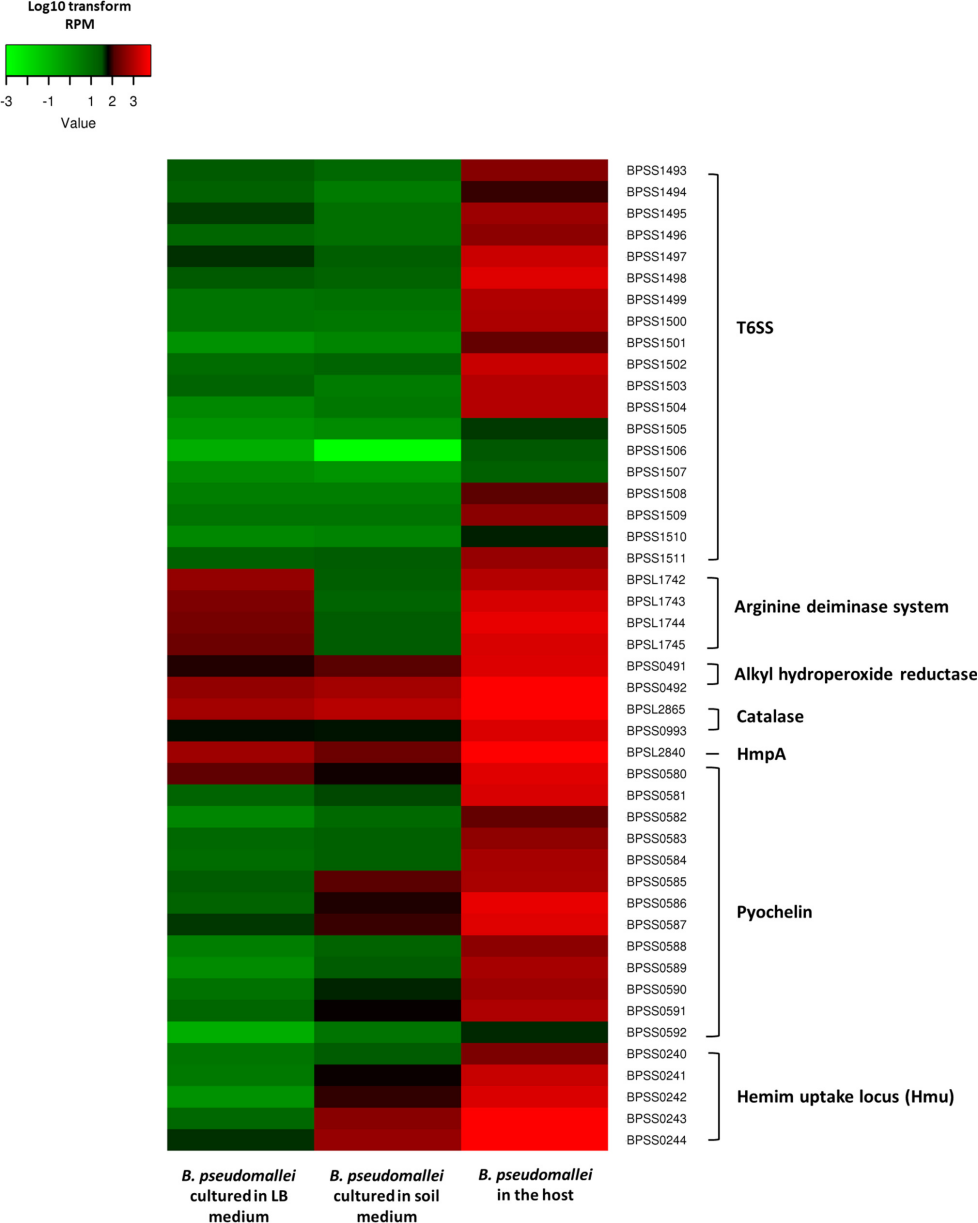
Heat map comparing UKMD286-induced transcriptional responses of selected genes in SEM, LB medium, and host. Colors symbolize transformed FPKM counts, with green representing low expression, black representing intermediate expression, and red representing high expression.

We next asked whether the positive regulation of B. pseudomallei virulence factors or defense-associated proteins when the bacteria are in a host truly reflected a change during the shift from soil to a host. We compared the expression profiles of selected genes between B. pseudomallei grown in SEM, in the host, and under laboratory conditions (cultured in LB medium). The expression profiles of these selected genes in B. pseudomallei cultured in LB or soil media were relatively similar (Fig. 4). These genes were negatively regulated in both media compared to that in bacteria during host infection. We did note that genes involved in the arginine deiminase system were enriched in LB medium compared to SEM.

**FIG 5:**
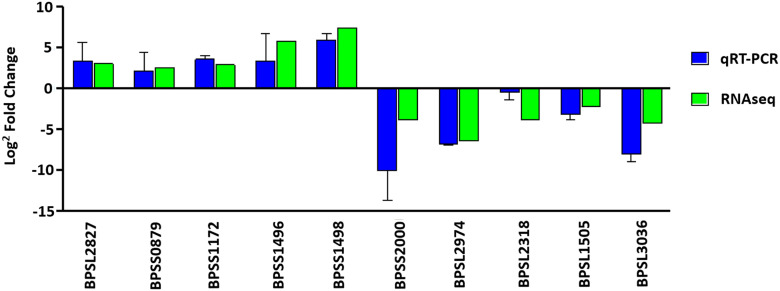
Verification of RNA sequencing data by quantitative real-time PCR (qRT-PCR). The graph shows relative expression ratios for qRT-PCR (blue bar) and RNA sequencing analysis (green bar) of 10 selected genes. Expression of the selected genes was normalized to the expression value for 23S rRNA. Vertical axis represents the log_2_ scale of the fold change. Values are averages of results from three independent biological replicates ± standard deviation.

The comparative expression profile obtained through RNA sequencing was verified by quantitative real-time PCR (qRT-PCR). For this analysis, we randomly selected a total of 10 genes that were either highly or lowly expressed during infection. The genes *bpsl2827* (dnaK), *bpss0879* (porin protein), *bpss1172* (polyethylene synthase), *bpss1496* (hypothetical protein), and *bpss1498* (hcpI) were selected to represent the highly expressed genes, while *bpss2000* (hypothetical protein), *bpsl2974* (lipoprotein), *bpsl2318* (glnA), *bpsl1505* (rpoS), and *bpsl3036* (outer membrane porin) were selected to represent genes exhibiting reduced expression during infection (highly expressed under soil-like conditions). Average cycle threshold values (*C_T_*) for all genes were normalized to 23S rRNA reference genes, indicating consistent expression for all conditions. Differential gene expression was determined using the 2^–ΔΔ^*^CT^* method and presented as fold change. The multiplier value was converted to log_2_ to enable direct comparison of the fold change for qRT-PCR and RNA sequencing-derived analysis. Similar profiles of the fold-change direction (up- or downregulated) were observed, albeit with different degrees of fold change (Fig. 5).

### Nitrogen metabolism and type III secretion system-2 are important for *B. pseudomallei* when it is present in the soil.

As noted previously, a total of 959 B. pseudomallei genes were expressed at least 2-fold higher (*P* < 0.01) when the bacteria were grown under soil conditions relative to their expression levels in bacteria isolated from infected spleens (Supplemental File 2, Data Set 2). From the KEGG pathway analysis (Table 2), we noted that B. pseudomallei genes involved in nitrogen metabolism were significantly enriched. Under anaerobic soil conditions, the nitrate reductase mechanism is important for bacteria to acquire nitrate from the soil and transport it into the cell, where the accumulated nitrate is converted to nitrite and then to ammonia (26). B. pseudomallei is abundant in disturbed soil that contains elevated levels of nitrates and total nitrogen (27).

Among the B. pseudomallei proteins that mapped to the nitrogen metabolism pathway, BPSS1163 is a fumarate/nitrate reduction family regulator (FNR), BPSS1244 is the nitrate:nitrite antiporter NarK, and BPSL2829 is a nitrogen transporter. The nitrate reductases encoded by the NarGHI cluster (BPSS1156, BPSS1159, and BPSS1163) are most likely highly expressed to convert accumulated nitrate to nitrite, an electron acceptor (12), to generate ammonium via the set of NirBD proteins (BPSS1242, BPSS1243, BPSL0511, and BPSL0512) (28). We also noted that urea-transport (UrtABCDE) genes (*bpsl2651* to *bpsl2655*) were also highly expressed. Under limited nitrogen concentrations in the soil, urea can be converted to ammonia by urease enzymes. The B. pseudomallei genes that encode the UreABCF urease (BPSL2657 to BPSL2659) were also positively regulated under soil-like conditions. The overexpression of BPSL2318 and BPSS0469, annotated as putative glutamine synthases (GlnA), may be involved in the conversion of ammonia into l-glutamine, a central reservoir of nitrogen for many biosynthetic pathways in bacteria, to ensure B. pseudomallei survival in the soil. Nonetheless, we show that these proteins are not required during active infection of a host.

**FIG 6:**
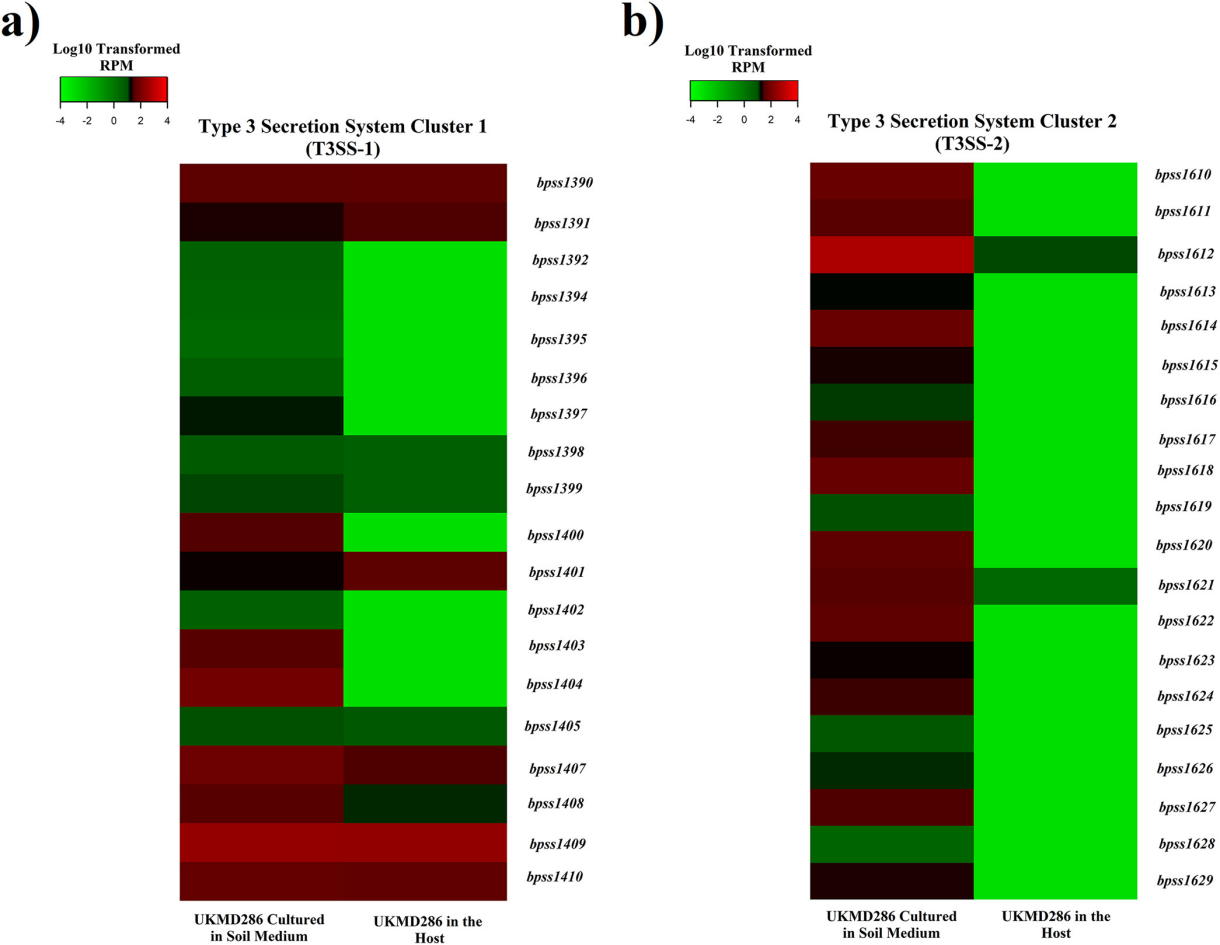
Heat map comparing the expression of B. pseudomallei UKMD286 genes involved in the type III secretory system of clusters (a) 1 (T3SS-1) and (b) 2 (T3SS-2). Color scale shows the value of the modified reads per million (RPM) gene expression. Green indicates low gene expression while red indicates high gene expression.

There are three types of type III secretion systems (T3SS) present in B. pseudomallei. T3SS-3 has been linked to mammalian virulence, while T3SS-1 and T3SS-2 have unknown functions but play minor roles in animal virulence (29, 30). In this study, the majority of T3SS-2 protein-encoding genes, including transcription regulator BpspB2 (BPSS1610), primary needle component (SctF, BPSS1612), and other secretion system machinery proteins (BPSS1614, BPSS1617, BPSS1618, BPSS1620, BPSS1622, BPSS1624, BPSS1627, and BPSS1629) were stimulated in the SEM environment but not during active infection of mice (Fig. 6). Ooi et al. (16) previously reported the activation of T3SS-2 genes in a nutrient-limited environment, but T3SS-2 does not appear to be required for the bacterium to escape from vacuoles into the cytoplasm of infected macrophages (29) and is not required for infection in a Syrian hamster model (31). Heacock-Kang et al. (32) also did not observe significant expression of the T3SS-2 system in B. pseudomallei isolated from infected cell lines. It is possible that the B. pseudomallei T3SS-2 evolved to help the bacterium avoid or survive predation by the many organisms which inhabit the soil and natural bodies of water, which may explain its overexpression under soil-like conditions.

### Distinct *B. pseudomallei* transcriptome landscape during an active *in vivo* infection.

**FIG 7:**
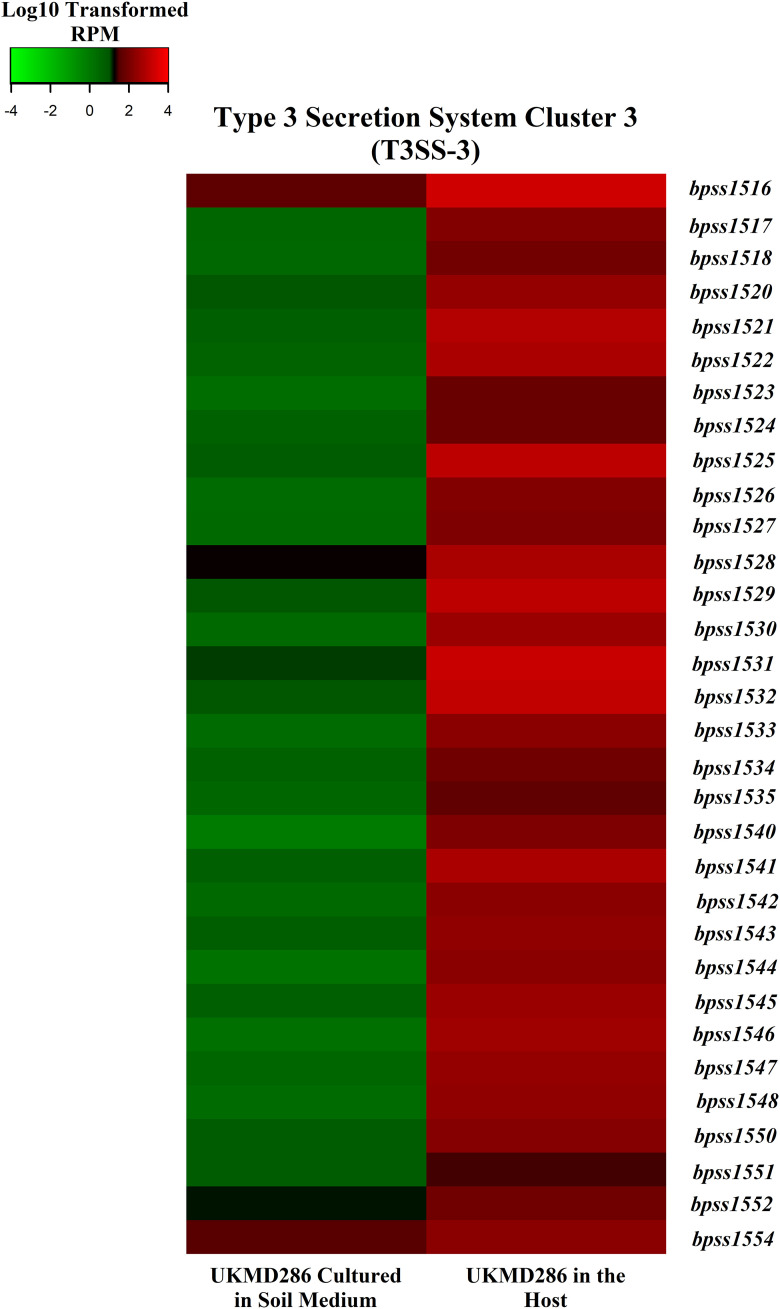
Expression of B. pseudomallei UKMD286 genes involved in the type III secretory system of cluster 3 (T3SS-3) were significantly overexpression on day-5 postinfection. Color scale shows the value of the modified RPM gene expression. Green indicates low gene expression while red indicates high gene expression.

A total of 475 B. pseudomallei genes were upregulated in bacteria isolated from infected spleens relative to the expression profile of bacteria cultured in SEM (Supplemental File 1, Data Set 3). These included genes which encode several B. pseudomallei virulence factors such as BimA, flagella, TssM, type III secretion system cluster 3 (T3SS-3), and type VI secretion system-1 (T6SS-1). KEGG pathway analysis of B. pseudomallei UKMD286 in a host environment also highlighted the enrichment of virulence factors and metabolic pathways (Table 3). B. pseudomallei is a facultative intracellular pathogen which exploits host cells such as macrophages and neutrophils to elude the immune system. B. pseudomallei uses BimA (BPSL1492) to rearrange the host actin polymer chain within an infected host cell, allowing the bacterium to move about (33) through the formation of multinucleated giant cells (MNGC) (34, 35). In mammalian hosts, T6SS and T3SS-3 are important B. pseudomallei virulence determinants. We noted that both secretion systems were induced during the active infection stage. T3SS-3 components were activated, including their effectors BopC (BPSS1516), BopA (BPSS1524), BipC (BPSS1531), and BipB (BPSS1532) (Fig. 7), which are essential for endocytic host cell invasion, vesicle escape, and MNGC formation (29, 36–39). BsaN (BPSS1546) regulates the two-component system VirAG (BPSS1495 and BPSS1494), which is responsible for the transcriptional regulation of T3SS and T6SS (40). T6SS-1 effector proteins, such as Hcp1 (BPSS1498), which are important for MNGC formation, play important roles during infection (41). Recently, Sanchez-Villamil et al. (42) identified virulence factors and regulatory proteins associated with T3SS-3 and T6SS-1 that are important for infection of intestinal epithelial cells. The authors noted that the BicA (BPSS1533) protein regulates both T3SS-3 and T6SS-1, as a Δ*bicA* mutant had reduced B. pseudomallei intracellular survival, reduced plaque formation, and impacted virulence in a murine model of gastrointestinal infection. In our study, BicA was also overly expressed during infection of a host compared to expression in soil conditions.

**TABLE 3:** Enriched KEGG pathway analysis of Burkholderia pseudomallei UKMD286 in the host environment

Functional category	KEGG pathway	Counts	Pop hits	*P*
Virulence factor	bps02040: Flagellar assembly	14	37	4.94E−06
Metabolism	bps01053: Biosynthesis of siderophore group nonribosomal peptides	5	7	1.15E−03
	bps00910: Nitrogen metabolism	9	39	1.53E−02
	bps00190: Oxidative phosphorylation	12	69	3.01E−02
Other functions	bps02020: Two-component system	21	137	1.05E−02

Unsurprisingly, genes encoding the different components of the B. pseudomallei flagellum (including *bpsl0270*, *bpsl0271*, *bpsl0273*, *bpsl0274*, *bpsl0275*, *bpsl0227*, *bpsl0031*, *bpsl0280*, *bpsl0281*, *bpsl3293*, and *bpsl3319*), were expressed at ~4-fold higher levels *in vivo* compared to that in SEM. In an infected host niche, the bacterial flagellum is crucial for adhesion to host cells as well as for early-stage colonization and tissue tropism (43). We noted that the flagellum components upregulated in the host were mainly structural components (filament, hook, etc.), while flagellum proteins required for bacterial movement (Motor/switch, MotA, etc.) were not enriched. Conversely, flagellum-driven swimming and swarming could be restricted in non-water-saturated soil where opportunities for dispersal are limited. TssM (BPSS1512), another B. pseudomallei virulence factor, was also induced during infection, similar to that seen in infected macrophages (44). TssM impairs NF-κB activation and type I interferon signaling; hence, the overexpression of B. pseudomallei TssM suggests the dampening of the host immunological response, leading to the high bacterial loads in the infected animals on day 5 postinfection.

We also noted that a number of known B. pseudomallei virulence factors were repressed on day 5 postinfection. Capsular polysaccharide inhibits phagocytosis by reducing C3b deposition on the cell surface (45), but also stimulates host immunological proteins such as MIG, RANTES, and interferon γ (IFN-γ) (46). As such, the reduced expression observed for BPSL2789 to BPSL2808 (key components of the capsular polysaccharide) during the active infection stage could be a strategy employed by the pathogen to prevent activation of the host immune response and prevent bacterial clearance. The repression of these genes was also noted for B. pseudomallei during infection of U937 human macrophage cells (20) and in a hamster model of melioidosis (19). Furthermore, quorum sensing was also repressed during infection. According to Horton et al. (47), attenuation of the *N*-acyl homoserine lactones (BPSS0885, BPSS1180, and BPSS1570) enhances MNGC formation. In conclusion, B. pseudomallei represses genes related to capsular polysaccharide and quorum sensing while inducing expression of the T6SS-1 during the active infection stage to avoid activating the host immune response.

### *B. pseudomallei* modifies the expression of different functional category genes under *in vivo* growth conditions.

The host uses ROS and reactive nitrogen intermediates (RNI) to destroy invading pathogens (48, 49). In this study, the proteins which protect the bacterium against host ROS and RNI were also upregulated, including the catalase enzymes KatG (BPSL2865) and KatB (BPSS0993) and the alkyl hydroperoxide reductases AhpF (BPSS0492) and AhpC (BPSS0491), to deactivate hydrogen peroxide (50, 51). AhpFC also protects bacteria from peroxynitrite (ONOO^–^), a highly reactive oxidant that plays a key role in the destruction of foreign pathogens by cells such as macrophages (52, 53). We noted the overexpression of the flavohemoprotein HmpA (BPSL2840), which converts nitric oxide (NO) to nitrous oxide, preventing the production of peroxynitrite from NO.

The arginine deiminase system (ADS) pathway involved in amino acid metabolism is known to protect bacteria from oxidative stress induced by the host (54, 55). When the bacteria transitioned from soil-like conditions into the host, we observed that the B. pseudomallei AcrABCD operon (BPSL1743 to BPSL1746) was activated. AcrD is a l-arginine/ornithine antiporter which transports l-arginine from the environment into the bacterial cell, where it is metabolized by AcrABC to produce ammonia (NH_3_), carbon dioxide (CO_2_), and ATP. While previous studies have proposed that the ADS may not be essential for bacterial survival or virulence, it may be required to protect the bacteria from oxidative damage to enable persistence or to permit morphotype switching over the course of infection (56, 57). The ADS in many bacteria, including B. pseudomallei, is also induced by iron limitation. The host maintains low levels of iron in the environment as part of its defense against bacterial invasion. However, B. pseudomallei circumvents this iron deficiency by triggering iron-scavenging malleobactin (BPSL1774 to BPSL1779) and pyochelin (BPSS0580 to BPSS0594) to harvest accessible iron in the surrounding environment (58). In addition, the hemin uptake loci, HMU (BPSS0240 to BPSS0244) and HEM (BPSL2720 to BPSL2724), which extract iron from the host’s heme protein, and TonB (BPSL2721 to BPSL2724), a membrane protein that helps bacteria transport sequestered iron (59), were also induced in the infected mice.

Increased temperature is a change consistently experienced by pathogens when they migrate from the external environment to a host. The transition from soil to a mammalian host exposes B. pseudomallei to changes in temperature and temperature-associated damage. Therefore, it was not surprising to note the induction of various heat shock-related proteins such as RpoH (BPSL0540), a transcription factor that regulates the production of heat shock proteins DnaJ (BPSL2826), DnaK (BPSL2827), GrpE (BPSL2829), GroEL (BPSL2697), GroES (BS2919), and ClpB (BPSL1484) (60, 61), as well as HtpG (BPSL1087), FtsH (BPSS1114), HslU (BPSL0203), and HslV (BPSL0203). Several drug transporters, including MacAB-OprJ (BPSS0623-BPSS0624) (62) and AmrAB-OprA (BPSL1802–BPSL1804), were also upregulated in bacteria isolated from infected spleens. Bacteria release secondary metabolites in response to environmental stress or host contact during infection to provide them with a competitive edge. During infection, the toxins malleilactone (BPSS0299 to BPSS0311) and bactobolin (BPSS1167 to BPSS1174) (63, 64), as well as CapBCD (BPSL1795 to BPSL1797), a Burkholderia thailandensis capistruin-like secondary metabolite, were induced. Capistruin-like secondary metabolite, a member of the lasso peptide secondary metabolite family, is an antibacterial agent that targets bacterial RNA polymerases (65, 66). The LysR-type transcriptional regulator ScmR is reported to negatively regulate the synthesis of malleilactone, bactobolin, and capistruin (67). From our analysis, the LysR-type transcriptional regulators are negatively regulated in bacteria during infection (Supplemental File 1), and this concurs with the report that B. pseudomallei ScmR mutants are hypervirulent in a Caenorhabditis elegans infection model (68). ScmR also suppresses expression of T3SS genes (68), and the induction of T3SS-3-related genes during *in vivo* infection, as noted previously, may also be attributed to the reduced expression of LysR-type transcriptional regulators (69). Generally, capistruin production is stimulated under stressful conditions; however, the role of the capistruin-like secondary metabolite during infection warrants further investigation.

### Differential regulation of *B. pseudomallei* genes during the early, active, and late phases of infection.

One limitation of this study is that the expression profiling of B. pseudomallei during an active infection of mice is limited to a single time point, 5 dpi, to represent the point at which bacterial loads were at the mid-log phase. Because extenuating factors prevented the dual-RNA sequencing strategy to be extended to other infection time points, qRT-PCR was adopted to analyze the expression of selected bacterial genes using total RNA from infected animals euthanized on days 3 (early infection) and 7 (late-stage infection).

**FIG 8:**
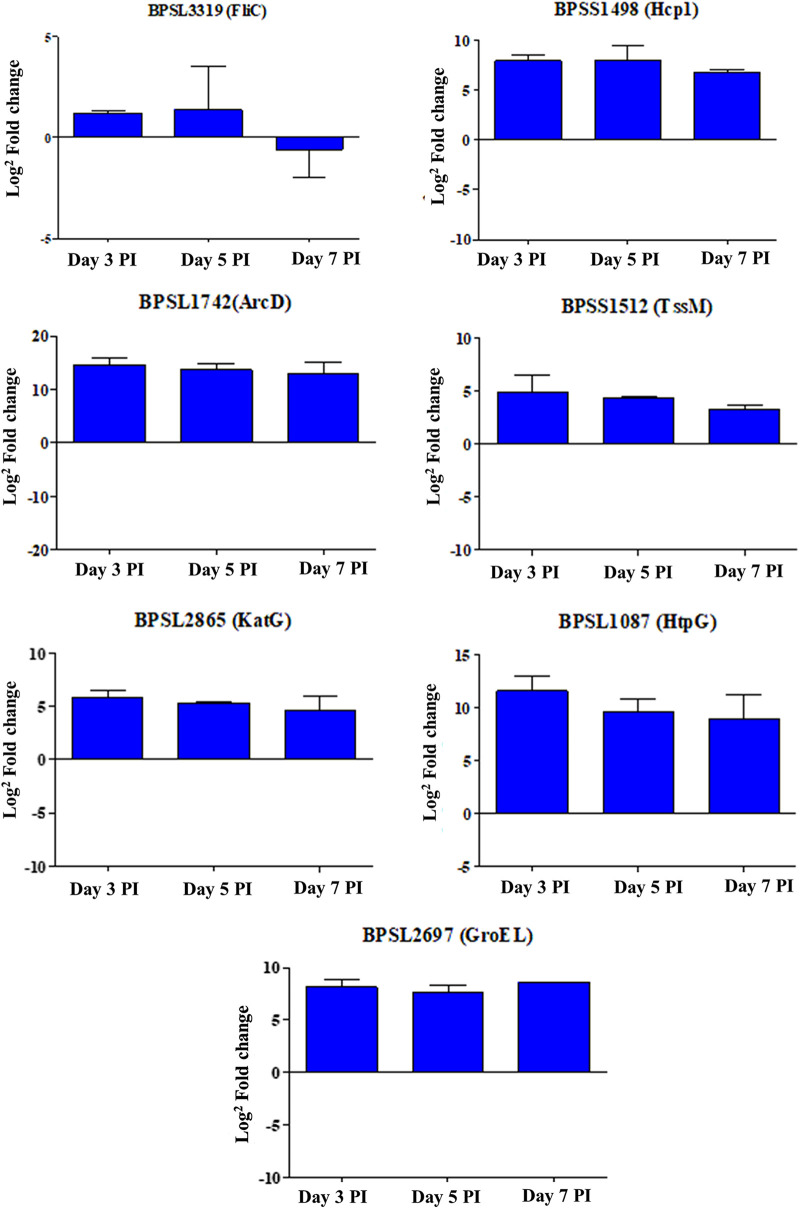
Expression profiles of selected UKMD286 genes in days 3, 5, and 7 postinfection.

Quantitative RT-PCR analysis was performed on B. pseudomallei genes involved in virulence, metabolism, and defense against the host immune system and stress which were overexpressed on day 5 postinfection. The expression profiles of *bpsl3319* (*fliC*), *bpss1498* (*hcpI*), *bpsl1742* (*AcrD*), *bpss1512* (*tssM*), *bpsl1087* (*HtpG* protease), *bpsl2987* (*GroEL*), and *bpsl2865* (*KatG*) on days 3, 5, and 7 postinfection are shown in Fig. 8. FliC, a major component of flagellin, was highly expressed on days 3 and 5 postinfection but decreased during the late phase of infection. The reduced expression on day 7 suggests the bacteria switched from the height of the infection phase to an intracellular growth phase during which B. pseudomallei changes from flagella-based motility to BimA-associated intracellular movement (33) to further avoid detection by the host immune system. The B. pseudomallei virulence factor TssM (*bpss1512*) is known to suppress the host immune response, and *bpss1512* was highly expressed from days 3 to 7 postinfection. The AcrD protein-encoding gene was also highly expressed at all time points, highlighting the importance of the arginine deiminase system in ensuring bacterial survival. The *bpsl1087* and *bpsl2987* genes, encoding HtpG protease and GroEL clamps, respectively, act to ensure protein functionality during stress. The *bpsl2865* gene encoding the KatG heme-peroxidase catalysis protein was also highly expressed at each phase of infection, indicating that high KatG expression assists B. pseudomallei in overcoming the detrimental effects of host-induced oxidative stress (49).

## DISCUSSION

**FIG 9:**
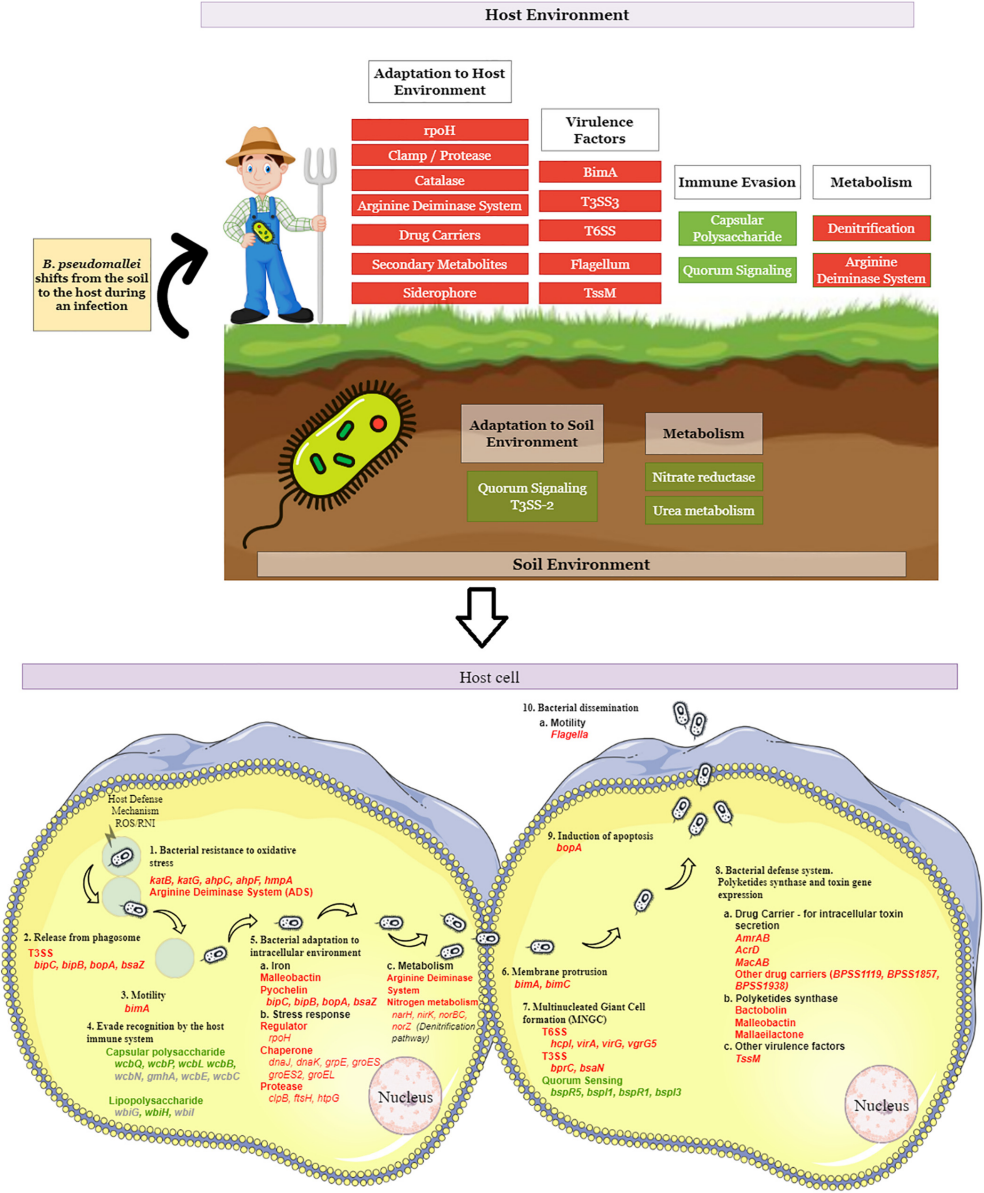
Major differences between the expression profiles in which a specific set of B. pseudomallei genes are highly expressed when the bacteria were cultured in soil extract medium, while a different set of genes identified encoded proteins that are important for B. pseudomallei survival and replication within the infected host. Green indicates low gene expression while red is for high gene expression.

Pathogenic bacteria need to rapidly adjust their virulence and fitness programs when they shift from one ecological niche to another. By monitoring infection-linked transcriptome alterations, virulence-related factors and regulatory processes that drive bacterial pathogenesis can be determined. A number of reports have previously identified transcriptome-level changes in B. pseudomallei as it adapts to different *in vitro* or *in vivo* (cellular) environments (reviewed in Yip et al. [70]). In this study, we set out to expand on existing knowledge by exploring how B. pseudomallei adjusts its physiology when it transitions from its natural soil environment into a mammalian host. Our initial approach to isolate live bacteria from infected mouse organs for bacterial RNA preparation was unsuccessful, and therefore we utilized a dual-RNA sequencing strategy in which total RNA from infected mouse spleens, enriched for bacterial mRNA, was sequenced and bacterial transcripts were compared to transcripts derived from B. pseudomallei cultured in a soil-like medium. We observed major differences between the expression profiles where a specific set of B. pseudomallei genes was highly expressed when the bacteria were cultured in soil extract medium while a different set of genes identified encoded proteins that are important for B. pseudomallei survival and replication within the infected host (Fig. 9).

B. pseudomallei is a facultative anaerobe that can survive in soils approximately 30-cm deep (27), where oxygen levels are low, such as in rice paddy fields. B. pseudomallei uses nitrate for nitrogen respiration in environments where oxygen is scarce, such as soil (71). In the B. pseudomallei genome, genes involved in nitrogen respiration are found throughout chromosomes 1 and 2, attesting to the importance of the associated proteins in ensuring bacterial adaptation to anoxic conditions (28). As noted above, nitrogen metabolism-related protein-encoding genes were induced in B. pseudomallei cultured under soil-like conditions (Fig. 3). When in the soil, the bacteria use a nitrate reduction pathway to perform anaerobic respiration and produce energy in the form of ATP. On the other hand, in the host environment, B. pseudomallei perform denitrification of nitrate to generate energy. We noted that B. pseudomallei employs distinct sets of nitrogen metabolism-related genes for growth in diverse surroundings, suggesting that different environmental cues and B. pseudomallei transcription factors or regulators may be involved in activating distinctive sets of genes under different conditions.

B. pseudomallei infection in the host takes place over three stages: (i) entry and uptake; (ii) survival, replication, and escape; and (iii) dissemination and MNGC creation (72). Our findings imply that by day 5 postinfection in mice, B. pseudomallei progressed from stage 1 to stages 2 and 3. This is supported by the observation that virulence factors required during entry and uptake, such as type IV pilin, BoaA, and BoaB, were either repressed or not regulated, whereas most protein-encoding genes involved in stages 2 and 3 were modulated. This suggests that when bacterial loads were highest in the animal, B. pseudomallei had successfully infiltrated intracellularly, adapted to the host cell environment, and was actively spreading infection via MNGC production. B. pseudomallei genes important for survival, replication, and escape, such as T3SS-3 and BimA, were modulated during the active infection phase. T3SS-3 effectors such as BopA, BsaQ, BipC, BipB, and BsaZ play a crucial role in phagosome escape, allowing the bacteria to enter the host cell cytoplasm and avoid being repeatedly exposed to the host’s damaging molecules (36, 73). In the host cell cytoplasm, large amounts of BimA-, BprC-, and VirAG-driven T6SS-1 may enable the bacteria to mobilize across the cytoplasm of their host (73).

Interestingly, during an active B. pseudomallei infection, the data hinted that both capsular polysaccharide biosynthesis and quorum sensing were suppressed. CPS, an essential B. pseudomallei virulence factor, constitutes the outermost layer of the cell that protects the bacteria from phagocytosis (45). Both Chieng et al. (20) and Tuanyok et al. (19) reported that CPS is not expressed during the early stages of host infection, suggesting that the CPS is not required at every stage of infection. The presence of CPS can activate the host immune response via interferon activation; hence, suppression of the polysaccharide conceals the presence of the bacteria from the host immunological response (46). Repression of quorum sensing, a critical pathogenic component, may have affected B. pseudomallei virulence (74, 75). Horton et al. (47) previously reported that B. pseudomallei do not utilize quorum sensing during intracellular growth, and AHL synthase-deficient B. pseudomallei could still form MNGCs. Thus, the repression of quorum sensing and the activation of T6SS may be the pathogen’s strategy to develop MNGCs and promote bacterial dissemination.

B. pseudomallei regulate oxidative stress, iron sequestration, and heat-shock stress to adapt to the host environment (76). In the infected host, B. pseudomallei trigger the production of catalase (KatG and KatB) and alkyl hydroperoxide reductase (AhpC and AhpF) to neutralize host-generated ROS and RNI and ensure their survival. A large number of B. pseudomallei genes involved in overcoming oxidative stress were expressed at extremely high levels, supporting the essentiality of this process for B. pseudomallei survival in the host environment (Supplemental File 1). Secondary metabolites also assist bacteria to adjust to environmental changes. We noted the overexpression of B. pseudomallei genes encoding the secondary metabolites malleilactone, bactobolin, and capistruin in the infected mice. We previously demonstrated that B. pseudomallei bactobolin disrupts the host translation machinery (77). Both malleilactone and bactobolin are antibiotics and known to be toxic to the host (63, 64); however, the role of capistruin during infection is unknown. Capistruin is a threaded-lasso peptide with a 9-amino-acid N-terminal ring and a 10-amino-acid C-terminal tail which threads through the ring (65). The secondary metabolite has antimicrobial activity and targets bacterial RNA polymerase to disrupt transcription in competing bacteria. Lasso peptide has been implicated in other bacterial infections. For example, bacteriocin, a member of the lasso peptide secondary metabolite family, is expressed by Streptococcus pneumoniae during infection (78). In this report, we focused on bacterial proteins with assigned functions; however, there are also a number of B. pseudomallei proteins of unknown function. Many of these proteins are conserved across other soil-dwelling pathogenic *Burkholderia* species, and assigning functions to these proteins would assist in delineating them as potential targets to control the bacterium.

In conclusion, there are detectable changes in the B. pseudomallei gene expression profile when the bacterium transitions from its natural environment (soil) to its mammalian host; however, whether these changes are also manifested during a typical infection of an individual after direct contact with B. pseudomallei-contaminated soil remains to be validated. Despite the limitations of the study, our findings reflect how soil-dwelling B. pseudomallei have the genetic capacity to adapt to a host after infection. The proteins that were detected as being overexpressed under infection conditions could serve as promising therapeutic targets.

## MATERIALS AND METHODS

### Bacterial strain and cultivation in soil medium.

The clinical isolate B. pseudomallei UKMD286 was used in this study. The bacteria were grown in soil extract medium (10% soil extract [prepared by autoclaving 400 g of sieved soil per L of water for 15 min, centrifuging, and filtering to remove particulates]) as described by Yoder-Himes et al. (21). Briefly, a single bacterial colony was cultured in 30 mL of brain heart infusion broth (BHIB) overnight at 37°C on a rotary shaker to create the inoculum for the SEM. The overnight inoculum was centrifuged for 10 min at 4,000 × *g* and the pellet was resuspended in 2 mL of spent supernatant. One mL of inoculum was added into 30 mL of SEM and incubated for 20 h at 29°C.

### Animals.

All animal studies were carried out in compliance with the animal ethical requirements of Universiti Kebangsaan Malaysia and were approved by the Universiti Kebangsaan Malaysia Animal Ethics Committee (UKMAEC; FST/2016/SHEILA/23-MAR./732-MAR.-2016-OCT.-2018). BALB/c mice aged 7 to 9 weeks were purchased from the University Animal Facility, Bangi, Malaysia. They were housed in individually ventilated cages made of high-temperature polysufone (Techniplast, Italy) with corncob bedding and a 12-h light/dark cycle. Mice were fed *ad libitum* with commercial pellets and purified water.

### Determination of *B. pseudomallei* UKMD286 50% lethal dose.

The LD_50_ for B. pseudomallei UKMD286 was assessed over a 10-day period or when at least 50% of the mice had died. BALB/c mice were divided into four groups of 10 mice each, and each group received a different dose of UKMD286 cultivated in SEM and delivered by intraperitoneal injection. The doses reported (10^−1^ to 10^−5^ CFU/mL) are the actual doses of the inoculums as determined by colony counts on Ashdown agar (see below). As a control, a set of five mice was given 200 μL of sterile phosphate-buffered saline (PBS). All mice were kept in pathogen-free environments and given access to food and drink *ad libitum*. After the inoculation, mice were monitored daily for signs of disease and mortality over a 10-day period (79).

### Enumeration of viable *B. pseudomallei* in the organs of infected mice.

B. pseudomallei UKMD286 cultivated in SEM was adjusted to a density corresponding to about 1.5 × 10^8^ CFU/mL (0.5 McFarland nephelometer standard). For inoculation, the suspension was diluted to 0.1× LD_50_ in a total volume of 0.2 mL. The suspension was administered intraperitoneally into each respective mouse group, and the actual number of bacteria inoculated was gauged by plating the suspension on Ashdown agar to determine colony counts after 48 h. At 0, 1, 3, 5, 7, 9, and 11 dpi, three mice were killed, and the liver, spleen, and kidney were aseptically removed and placed in a tube containing sterile PBS. A hand-held motorized homogenizer (IKA T10 Basic Ultra-Turrax, IKA, Germany) was used to homogenize the individual organs. Organ homogenates were diluted 10-fold in PBS and 100 μL of each dilution was plated on Ashdown agar. The number of bacteria was enumerated as CFU per organ.

### Mice infection and preparation of enriched bacterial RNA.

Once the optimal infection conditions were determined, a different set of BALB/c mice was infected with 0.1× LD_50_ bacteria grown in SEM, and on day 5 postinfection, mouse spleens were harvested for total RNA extraction. Spleens were removed aseptically and stored in RNAlater (Ambion, Austin, TX) at −80°C until RNA extraction was conducted. Total RNA was extracted from 50 mg of each spleen using TRIzol (Invitrogen, Carlsbad, CA), subjected to RNase-free DNase set (Qiagen, Hilden, Germany) treatment according to the manufacturer’s instructions and purified using the RNeasy Minikit (Qiagen) as directed by the manufacturer.

Bacterial RNA was enriched from the total RNA extracted from mouse spleens using a method adapted from Mavromatis et al. (80). To eliminate host total RNA as well as bacterial and host rRNAs, 25 g of the extracted mouse spleen total RNA was processed with a MICROBEnrich kit (Ambion) according to the manufacturer’s instructions. The total RNA was then treated with the Ribo-Zero Gold rRNA Removal kit (Epidemiology) (Illumina, San Diego, CA) as directed by the manufacturer, to remove leftover host or bacterial rRNA. The quality and quantity of RNA obtained following bacterial RNA enrichment and rRNA depletion were determined with an Agilent 2100 Bioanalyzer and a LabChip 6000 RNA kit (Agilent Technologies, Santa Clara, CA).

### Total RNA extraction from bacteria cultured in soil extract medium.

For total bacterial RNA isolation, B. pseudomallei UKMD286 was cultured in 50 mL of SEM at 29°C for 8 h until an optical density at 600 nm (OD_600_) of 0.8 was achieved. The bacterial culture was centrifuged at 10,000 × *g* at 4°C for 10 min. The supernatant was removed and replaced with 3 mL of TRIzol (Invitrogen,). Bacterial pellets were dissolved, and RNA extraction was performed according to the manufacturer’s instructions. Residual DNA was completely removed using the RNase-Free DNase Set (Qiagen) and complete removal was validated by PCR with B. pseudomallei *recA* gene primers. Total RNA (10 μg) was subjected to 23S and 16S rRNA removal using the MicrobExpress kit (Ambion). The quality and quantity of RNA extracted were examined using 1.5% agarose gel, a ND-1000 Nanodrop spectrophotometer (Agilent Technologies), and an Agilent 2100 Bioanalyzer (Agilent Technologies).

### RNA sequencing.

All RNA samples which fulfilled the following requirements were used for transcriptome sequencing: *A*_260/280_ > 1.8, *A*_260/230_ > 2.0, and RIN > 7, except for enriched RNA samples which had no accurate RIN value due to rRNA removal. The integrity of the enriched RNA samples was assessed by gel electrophoresis to ensure the absence of potential RNA degradation represented by low molecular weight smearing.

An Illumina TruSeq kit was used to prepare RNA-seq libraries in two biological replicates. The Illumina HiSeq4000 platform was used for sequencing using a 100-bp paired-end protocol over 100 cycles to sequencing depths of approximately 10, 100, and 50 to 200 million reads for RNA from B. pseudomallei UKMD286 cultured in SEM, RNA from mouse spleens, and enriched RNA from mouse spleens, respectively. Raw reads generated from the sequencing process were submitted to the European Nucleotide Archive (ENA) (E-MTAB-11200).

### Analysis of differential expression.

FastQC and FastXtool were used to pre-process sequence reads generated from the sequencing process, based on a minimum approved Illumina quality value of 20 and a minimum read size of 30 bp. Using a Python script, the pre-processed reads were further filtered by removing orphan reads. The processed reads were aligned to the B. pseudomallei UKMD286 genome (accession no. GCA_902141795.1) (23) and the mouse GRCm38 genome sequence (accession no. GCA_000001635.9) using Hisat 2 version 2.2.0 (81) with the default parameters. Mapping of the clean raw data to the respective reference genomes separated mouse GRCm38 and B. pseudomallei UKMD286 transcripts. The DESeq2 software in R was used for differential expression analysis (82). DAVID (83) was used to determine differentially expressed genes while WEGO (Web Gene Ontology Annotation Plot) was used to visualize enriched GO keywords (84).

### Quantitative Real-Time PCR.

To validate the RNA-seq analysis, qRT-PCR was performed on selected bacterial genes that were modulated when the bacterium was either in the host or grown in SEM. A total of 10 genes, representing 5 B. pseudomallei genes induced during growth in SEM and 5 genes induced during infection of the host, were selected to validate the RNA-seq analysis. Primer sets were designed using the Real-Time PCR Tool (Integrated DNA Technologies; https://www.idtdna.com/scitools/Applications/RealTimePCR/). The list of genes and associated primers are available in Supplemental File 2. Briefly, 20-μL reaction mixtures of 20 ng cDNA and 10 pmol of each primer were prepared according to the manufacturer’s instructions for the QuantiNova kit (Qiagen). The Bio-Rad CFX96 real-time PCR machine (Bio-Rad Laboratories, Hercules, CA) was used to perform qRT-PCR. Two biological samples were run with three technical replicates, no template controls and no RT enzyme controls. Average cycle threshold (*C_T_*) values of the three technical replicates for each gene of interest were normalized to 23S rRNA, and relative expression levels were calculated using the 2^−ΔΔ^*^CT^* method (85).

### Data availability.

The data sets of raw reads generated for this study can be found in the ENA (https://www.ebi.ac.uk/ena/browser/home) under the reference number E-MTAB-11200.
