# Correction for Ghazali et al., “Transitioning from Soil to Host: Comparative Transcriptome Analysis Reveals the *Burkholderia pseudomallei* Response to Different Niches”

**DOI:** 10.1128/spectrum.00522-24

**Published:** 2024-03-08

**Authors:** Ahmad-Kamal Ghazali, Mohd Firdaus-Raih, Asqwin Uthaya Kumar, Wei-Kang Lee, Chee-Choong Hoh, Sheila Nathan

## AUTHOR CORRECTION

Volume 11, no. 2, e03835-22, 2023, https://doi.org/10.1128/spectrum.03835-22. Page 19, Acknowledgments, first sentence: “the Ministry of Education of Malaysia (FRGS/1/2021/STG02/UKM/01/1)” should read “the Ministry of Higher Education of Malaysia (MOHE) through the Fundamental Research Grant Scheme (FRGS) under the grant number FRGS/1/2021/STG02/UKM/01/1.”

