# Supplementary material for: Transitioning from Soil to Host: Comparative Transcriptome Analysis Reveals the Burkholderia pseudomallei Response to Different Niches
Source: Microbiol Spectr. 2023 Mar 1;11(2):e03835-22. doi: 10.1128/spectrum.03835-22 (PMC10100664; doi:10.1128/spectrum.03835-22)
Supplement: Supplemental file 2 — Data Set S2. Download spectrum.03835-22-s0002.pdf, PDF file, 0.1 MB [file spectrum.03835-22-s0002.pdf]

Supplementary File 2: Primers used for quantitative real-time PCR

Genes	Primer orientation	Primer sequence (5’ to 3’)
bpsl1505	Forward	CGA CAA TGA ACG CGA CTA CG
	Reverse	ATA GCC TTT CGC GAT GCT GA
bpsl2318	Forward	TCG AGA ACT TCG TGC CGA TT
	Reverse	CCG GAG CCG GAA TTC TTC AT
bpsl2827	Forward	CGC AGA TCG AAG TGA CCT T
	Reverse	ATC TTC TCG ATC TCG GCT TC
bpsl2974	Forward	AAG CGC AGC TGT TAT TAG CC
	Reverse	ATG TTG CCC GAA CTG TTC TC
bpsl3036	Forward	GCC CAT ACG TAC CAG GTG AT
	Reverse	GAA GTT GAT TTC CGC GTT GT
bpss0879	Forward	CAT CAC GTA CCA AAG CAA CG
	Reverse	GCT TTC CAA CGT GAA GAT CG
bpss1172	Forward	GGC TGT CCT ATC TGC TCG AC
	Reverse	GGC GAG AAG AAA CTG TCC TG
bpss1496	Forward	AGC GGG TCA ACA TCG TCT AT
	Reverse	ACG TCG TTG AAG TCG TCC TT
bpss1498	Forward	GTC ATG ACG GGA AAA TCC AC
	Reverse	GTC ATG ACG GGA AAA TCC AC
bpss2000	Forward	ACA GCC TGA CGC TCA AGA AT
	Reverse	TGT AGA CGT CGA CCT GCT TG
23s rRNA	Forward	GTA GAC CCG AAA CCA GGT GA
	Reverse	CAC CCC TAT CCA CAG CTC AT
